# MYC Overexpression Induces Prostatic Intraepithelial Neoplasia and Loss of Nkx3.1 in Mouse Luminal Epithelial Cells

**DOI:** 10.1371/journal.pone.0009427

**Published:** 2010-02-25

**Authors:** Tsuyoshi Iwata, Denise Schultz, Jessica Hicks, Gretchen K. Hubbard, Laura N. Mutton, Tamara L. Lotan, Carlise Bethel, Matthew T. Lotz, Srinivasan Yegnasubramanian, William G. Nelson, Chi V. Dang, MengMeng Xu, Uzoma Anele, Cheryl M. Koh, Charles J. Bieberich, Angelo M. De Marzo

**Affiliations:** 1 Department of Pathology, The Johns Hopkins University School of Medicine, Baltimore, Maryland, United States of America; 2 Department of Urology, The Johns Hopkins University School of Medicine, Baltimore, Maryland, United States of America; 3 Department of Oncology, The Johns Hopkins University School of Medicine, Baltimore, Maryland, United States of America; 4 The Sidney Kimmel Comprehensive Cancer Center at Johns Hopkins, The Johns Hopkins University School of Medicine, Baltimore, Maryland, United States of America; 5 The Brady Urological Research Institute, The Johns Hopkins University School of Medicine, Baltimore, Maryland, United States of America; 6 Department of Hematology and Medicine, The Johns Hopkins University School of Medicine, Baltimore, Maryland, United States of America; 7 Department of Biological Sciences, The University of Maryland Baltimore County, Baltimore, Maryland, United States of America; Uppsala University, Sweden

## Abstract

Lo-MYC and Hi-MYC mice develop prostatic intraepithelial neoplasia (PIN) and prostatic adenocarcinoma as a result of MYC overexpression in the mouse prostate[1]. However, prior studies have not determined precisely when, and in which cell types, MYC is induced. Using immunohistochemistry (IHC) to localize MYC expression in Lo-MYC transgenic mice, we show that morphological and molecular alterations characteristic of high grade PIN arise in luminal epithelial cells as soon as MYC overexpression is detected. These changes include increased nuclear and nucleolar size and large scale chromatin remodeling. Mouse PIN cells retained a columnar architecture and abundant cytoplasm and appeared as either a single layer of neoplastic cells or as pseudo-stratified/multilayered structures with open glandular lumina—features highly analogous to human high grade PIN. Also using IHC, we show that the onset of MYC overexpression and PIN development coincided precisely with decreased expression of the homeodomain transcription factor and tumor suppressor, Nkx3.1. Virtually all normal appearing prostate luminal cells expressed high levels of Nkx3.1, but all cells expressing MYC in PIN lesions showed marked reductions in Nkx3.1, implicating MYC as a key factor that represses Nkx3.1 in PIN lesions. To determine the effects of less pronounced overexpression of MYC we generated a new line of mice expressing MYC in the prostate under the transcriptional control of the mouse *Nkx3.1* control region. These “Super-Lo-MYC” mice also developed PIN, albeit a less aggressive form. We also identified a histologically defined intermediate step in the progression of mouse PIN into invasive adenocarcinoma. These lesions are characterized by a loss of cell polarity, multi-layering, and cribriform formation, and by a “paradoxical” increase in Nkx3.1 protein. Similar histopathological changes occurred in Hi-MYC mice, albeit with accelerated kinetics. Our results using IHC provide novel insights that support the contention that MYC overexpression is sufficient to transform prostate luminal epithelial cells into PIN cells *in vivo*. We also identified a novel histopathologically identifiable intermediate step prior to invasion that should facilitate studies of molecular pathway alterations occurring during early progression of prostatic adenocarcinomas.

## Introduction

Prostate cancer proceeds through a morphological progression consisting of the development of prostatic intraepithelial neoplasia (PIN), invasive adenocarcinoma, distant metastatic disease, and androgen refractory metastatic disease [2], [3], [4], [5]. The key morphological features that are diagnostic of PIN and invasive carcinoma cells are changes in nuclear morphology such as enlargement of the nucleus, changes in chromatin structure, and marked nucleolar enlargement [6], [7], [8], [9]. A critical issue remaining in cancer biology, including in prostate cancer, is determining the phenotype and niche of the progenitor cell that becomes transformed. Prostate epithelium consists of two distinct cell types, basal and luminal. While there is still controversy in this field, compelling evidence in both mice and humans suggests that normal prostate epithelial tissue stem cells reside in the basal compartment [10], [11], [12], [13], [14], [15], [16]. Since PIN cells have a luminal cell-like phenotype and are topographically present within the luminal compartment [7], [17], [18], [19], [20], we and others have suggested that cells within the luminal compartment, perhaps with features of both basal and luminal cells, are the targets for neoplastic transformation [19], [20], [21], [22]. Further, only luminal cells have somatic telomere shortening, which is highly characteristic of human PIN and adenocarcinoma[20]. We have postulated that atrophic epithelial cells, that often occur in large numbers in regions of inflammation (e.g. proliferative inflammatory atrophy/PIA), are often the target cells for transformation. This hypothesis is based upon morphological transitions of atrophy to PIN, and at times directly to micro-invasive adenocarcinoma, and rare molecular alterations such as methylation of *GSTP1* [23] and telomere shortening [24]. Another possibility was recently revealed by Wang et al., who showed that rare cells within the luminal compartment of the mouse prostate, that express Nkx3.1 in an androgen independent fashion (referred to as castrate resistant Nkx3.1 expressing cells or CARNs), possess stem cell characteristics and can be a target of neoplastic transformation[25]. But how do these aberrantly proliferating atrophic cells, or other luminal-like cells in non-atrophic epithelium, undergo transformation? The answer, at least in a significant fraction of cases, may relate to MYC expression (Note that the official gene name for what is commonly referred to as C-MYC is *MYC*).

MYC is an oncogenic transcription factor overexpressed in a variety of tumor types [26]. In prostate cancer, since a region encompassing the MYC locus (8q24) is amplified in late-stage/aggressive tumors, it is widely held that MYC is involved in disease progression [26], [27], [28], [29]. Yet, a number of observations suggest MYC may be involved in early phases of prostate cancer development as well. For example, MYC mRNA is overexpressed in the majority of all primary human clinical prostate cancer lesions [30], [31]. Further, marked overexpression of MYC protein occurs in the nuclei of the majority of primary prostatic adenocarcinomas, as well as, exclusively within the luminal compartment of high-grade PIN lesions, the presumptive precursor to many prostatic adenocarcinomas [31]. Together with the findings that overexpression of MYC in the mouse prostate causes PIN [1], [32] that progresses to adenocarcinoma[1], and that MYC can transform isolated human prostate epithelial cells into tumorigenic cells when mixed with urogenital sinus mesenchyme [33], these results suggest MYC overexpression may also be a critical factor contributing to prostatic adenocarcinoma initiation.

The mechanisms by which MYC transforms cells have been linked to its function as a nuclear transcription factor whereby it regulates a large number of genes and pathways that control a complex array of cellular processes including cell cycle progression, metabolism, ribosome biogenesis, protein synthesis, mitochondrial number and function, and stem cell self renewal [34], [35], [36]. In addition, MYC overexpression can result in global modifications to chromatin structure, which also may be critical for its ability to transform cells [37], [38], [39]. Given that a large number of potential changes induced by MYC may be required for transformation, and that some genes and pathways are regulated by MYC in a lineage/cell-type specific manner [36], it is important to determine both the nature and timing of molecular changes induced by MYC in specific target cell types *in vivo*. While these studies can be performed in human tissues to a certain extent, the inability to sample the human disease at multiple time points during progression of PIN lesions precludes precise determination of these events in humans. Genetically engineered mouse models that “phenocopy” the human disease can help decipher MYC's role in transformation and progression in a given lineage/cell type [36], [40].

Hi-MYC and Lo-MYC mice are transgenic mouse models that use either a portion of the rat probasin promoter (Lo-MYC mice), or the modified ARR_2/_ *probasin* promoter (Hi-MYC mice), to overexpress human *MYC* in a prostate-specific manner [1]. These mice develop PIN by 2 weeks (Hi-MYC) or 4 weeks (Lo-MYC) of age and invasive adenocarcinoma of the prostate by 6 to 9 months (Hi-MYC), or by 10–12 months (Lo-MYC). The phenotypes of the Hi- and Lo-MYC mice share a number of similarities with the human disease. For example, the histological features of PIN in Lo-MYC and Hi-MYC mice recapitulate stereotypical findings in human PIN and adenocarcinoma cells—atypical changes in nuclear morphology including enlargement of the nucleus and of nucleoli [1] (and see below). In addition, the phenotype of the cancer lesions in these MYC-based models is exclusively adenocarcinoma, with no evidence for the neuroendocrine carcinoma phenotype observed in tumor models based upon T antigen overexpression (e.g. TRAMP and LADY) [41], [42], [43], [44]. Furthermore, microarray expression profiling studies defined a gene expression signature of MYC-induced prostate cancer in Hi-MYC mice that shares a number of features with human prostate cancer [1], [45]. While these findings have been important for our understanding of the potential role of MYC in early human prostate cancer formation, additional studies are needed to address a number of enduring questions regarding MYC action in early prostate neoplasia and its relevance to the human disease.

One of the remaining issues regarding the early stages of neoplastic transformation is whether overexpression of MYC alone is sufficient for transformation to occur. The outcome of deregulated MYC expression is thought to depend on the overall levels of expression. Low levels of MYC expression, which occurs physiologically during cell proliferation in most cell types, is generally tolerated by cells without engaging tumor suppressor mechanisms, whereas high levels of MYC expression generally induce apoptotic and other tumor surveillance pathways [36], [46], [47], [48]. This indicates that in some cell types MYC needs to cooperate with other survival genes, which may or may not be classical activated oncogenes or inactivated tumor suppressors, to transform cells [36]. Further, ectopic MYC overexpression in some contexts may also cause cell cycle arrest [49] (e.g. human fibroblasts) or even terminal differentiation[50] (e.g. human keratinocytes). In other cell types, however, including prostate epithelial cells, MYC appears capable of immortalizing these cells *in vitro* in a single step without an apparent need for additional activated oncogenes or inactivated tumor suppressor genes [33], [51].

Another key question is whether changes in expression of a number of genes known to be relevant to prostate cancer may be explained, at least in part, by MYC overexpression. For example, Nkx3.1 is a lineage-restricted phenotypic transcription factor required for proper prostate development and secretory function [52], [53], [54], [55] which is often reduced in human prostate cancer and PIN lesions [56], [57], [58]. The mechanism of loss of Nkx3.1 in carcinoma is often related to loss of one allele of chromosome 8p [58], [59], and we have verified this finding in prostatic adenocarcinoma lesions [57]. In our recent study, however, most human PIN lesions showed reduced NKX3.1 protein expression without concomitant loss of chromosome 8p, as assessed by fluorescent in situ hybridization (FISH) [57]. This finding in human PIN suggests an additional mechanism, unrelated to chromosome 8p loss, for Nkx3.1 reduction in some prostate neoplastic lesions. In the present study we sought to determine in more detail by immunohistochemistry the relation between the onset of MYC accumulation and changes in Nkx3.1 protein and mRNA levels and the dynamics of such changes during disease progression. Our results suggest a new mechanism for Nkx3.1 loss in PIN and early prostate cancer.

Invasion of epithelial cells through the basement membrane into the stromal compartment is the pathognomonic event that underlies the diagnosis of epithelial cancers (e.g. carcinomas). The multistep process of invasive cancer formation reflects a complex series of events, and little is known regarding the transition states between the clearly preinvasive lesions, such as PIN, and the fully invasive adenocarcinoma lesions. In this study, we also examined the interface between PIN lesions and invasive adenocarcinoma lesions in MYC driven mouse models and we have identified a novel histopathologically definable intermediate step in the progression of PIN to micro-invasive carcinoma. This finding should prove useful for studies that delineate the molecular and cellular and micro-environmental processes of epithelial cell invasiveness.

## Materials and Methods

### Animal Husbandry

The experimental protocol was approved by the Animal Care and Use Committee at Johns Hopkins University, and the animals were cared for in accordance with institutional guidelines. The transgenic mice used in this study (Lo-MYC and Hi-MYC mice) were obtained from the Mouse Repository of the National Cancer Institute Mouse Models of Human Cancer Consortium at NCI Frederick, MD, USA. Lo and Hi-MYC mice are on the FVB background. Mice were housed in an animal facility maintained on a 12-h light/dark cycle, at a constant temperature (22±2°C) and relative humidity (55±15%). Tap water and food were available *ad libitum*. Hemizygous Lo-MYC mice on FVB background were cross-bred with non-transgenic FVB breeders. All control mice used at each time point were from FVB littermates. *Nkx3.1-MYC* transgenic mice were generated by pronuclear injection of single-cell FVB embryos essentially as previously described [60].

### Genotyping

Mouse-tail DNA was isolated using the DNeasy Blood & Tissue Kit from QIAGEN (Valencia, CA) and subjected to a PCR-based screening assay for genotyping. For Lo-MYC mice, primers were selected for genotyping using the human MYC cDNA sequence as follows: upstream primer, 5′-CAAGACTCCAGCGCCTTCTC-3′ and downstream primer 5′- AGCCTGCCTCTTTTCCACAG-3′, which resulted in a PCR product of 186 base pairs. For genotyping Hi-MYC mice, the upstream primer (located within the ARR_2_-PB promoter), 5′AAACATGATGACTACCAAGCTTGGC-3′ and the downstream primer (within the MYC cDNA sequence) 5′ATGATAGCATCTTGTTCTTAGTCTTTTTCTTAATAGGG-3′ were used to generate a PCR product of 177 base pairs. *Nkx3.1-MYC* transgenic founders were identified by Southern blot analysis of Bam HI digested genomic DNA using a probe within exon 2 of *Nkx3.1*.

### Histology

Mice were sacrificed by CO_2_ asphyxiation at various ages up to 24 months. The prostate lobes were dissected into ventral prostate (VP), dorso-lateral prostate (DLP) and anterior prostate (AP), and processed for histological and immunohistochemical analysis. In addition, the seminal vesicle, ampullary gland, lung, liver, kidney, spleen, colon and draining lymph nodes were immediately dissected for histological analysis. All mice were also examined carefully at necropsy for evidence of gross metastatic disease in all major organs except the brain. The tissues were fixed in 10% buffered formalin and examined by routine light microscopy after hematoxylin and eosin (H&E) staining.

### Antibodies

The anti-MYC antibody (rabbit monoclonal, clone Y69) was from EPITOMICS (Burlingame, CA). The anti-Nkx 3.1 antibody (rabbit polyclonal) was generated by the Bieberich lab [61]. Anti-Androgen receptor (AR) antibody (rabbit polyclonal) was from Santa Cruz Biotechnology (Santa Cruz, CA). The anti-Ki-67 antibody (rabbit polyclonal) was from Novocastra (Newcastle-upon-Tyne, United Kingdom), and the anti-cleaved caspase 3 antibody (rabbit polyclonal) was from Cell Signaling Technology (Danvers, MA). The anti-smooth muscle actin (SMA) antibody (mouse monoclonal, clone 1A4) was from DAKO Cytomation (Carpinteria, CA). The anti-β actin antibody (mouse monoclonal) was from Sigma-Aldrich (St. Louis MO).

### Immunohistochemistry

For antigen retrieval, slides were steamed for 40 minutes in EDTA solution (Zymed, South San Francisco, CA) for MYC and Nkx3.1. Slides were steamed for 40 minutes in DAKO Antigen Retrieval Solution for AR, Ki67, cleaved caspase 3, and SMA. Primary antibodies were incubated at the following dilutions: 1∶300 for MYC, 1∶200 for Nkx3.1, 1∶250 for AR, 1∶4800 for Ki67, 1∶50 for cleaved caspase 3, and 1∶1000 SMA. DAKO Catalyzed Signal Amplification kit was used for MYC. DAKO EnVision+ detection system was used for Nkx3.1, AR, Ki67, cleaved caspase 3, and SMA. Staining was visualized using 3,3′-Diaminobenzidine (DAB) (Sigma, Saint Louis, MO, FAST 3,3′-Diamino benzidine Tablets) and slides were counterstained with hematoxylin.

### Quantification of Immunostaining

Whole slides were scanned with the Aperio Scanscope CS at 200× magnification and random fields were selected for quantification using the open source software program, FrIDA, as described [31].

### Northern Blots

Total RNA (3 µg) was extracted from mouse ventral prostates and separated on 1% glyoxal/DMSO/agarose gels (Ambion Inc., Austin, TX) and hybridized to either a human *MYC* or mouse *Nkx3.1* cDNA probe. The hybridization signals were normalized with respect to the hybridization signal observed when the same blots were probed with a β-actin cDNA probe.

### Western Blots

Mouse ventral prostates were lysed in RIPA buffer. Equal amounts of total protein were electrophoresed and transferred to PVDF membranes for immunoblotting. Membranes were probed with antibodies against MYC (Epitomics, 1∶5000), Nkx3.1 (1∶1000) and β-actin (Sigma, 1∶10,000) (as a control to demonstrate equal protein loading). The membranes were incubated overnight at 4°C followed by 1 hour of incubation at room temperature with the secondary horseradish peroxidase-conjugated Donkey polyclonal anti-rabbit antibody (1∶5000 dilution, Amersham, Piscataway, NJ, USA for MYC and Nkx3.1) or anti-Mouse IgG, HRP-Linked Whole Ab from sheep (1∶5000 dilution, GE Healthcare NA931) for actin. Proteins were visualized using an ECL chemiluminescence detection system, following the manufacturer's protocol and Hyperfilm ECL (Amersham).

### Generation of the Nkx3.1-MYC Transgene

To create the *Nkx3.1-MYC* transgene, a recombineering targeting vector was generated consisting of the human MYC open reading frame and a Kanamycin^r^ gene flanked by 5′ and 3′ Nkx3.1 homologous arms [61]. The targeting fragment was removed from the vector and used to transform an E. coli SW102 strain [62] carrying the NK17/lacZ transgene [61] in the pClasper shuttle vector [63]. Recombinants selected for Kanamycin resistance were screened for the presence of the MYC gene by restriction enzyme analysis. A clone with the expected band pattern indicating that the MYC gene had correctly inserted into the Nkx3.1 locus was sequenced to ensure that precise insertion had occurred. In the final *Nkx3.1-MYC* transgene, transcription initiates at the Nkx3.1 transcriptional start site and polyadenylation is signaled by the SV40 early poly(A) sequence. Translation initiates at the Nkx3.1 start codon, proceeds through codons 2–6 of Nkx3.1 and three codons generated by cloning (GGG, glycine; GAT, aspartate; CCA, proline), then continues from codon 2 through 438 of MYC. Translation is terminated by the MYC TAA sequence.

### Statistics

Comparisons between the extent of nuclear staining for Ki67 between tissue types were evaluated using the Kruskal-Wallis test for equality of populations using Stata 8.0.

## Results

### Morphological Features of PIN Lesions in Lo-MYC Mice Are Highly Similar to Human High Grade PIN

**Figure 1:**
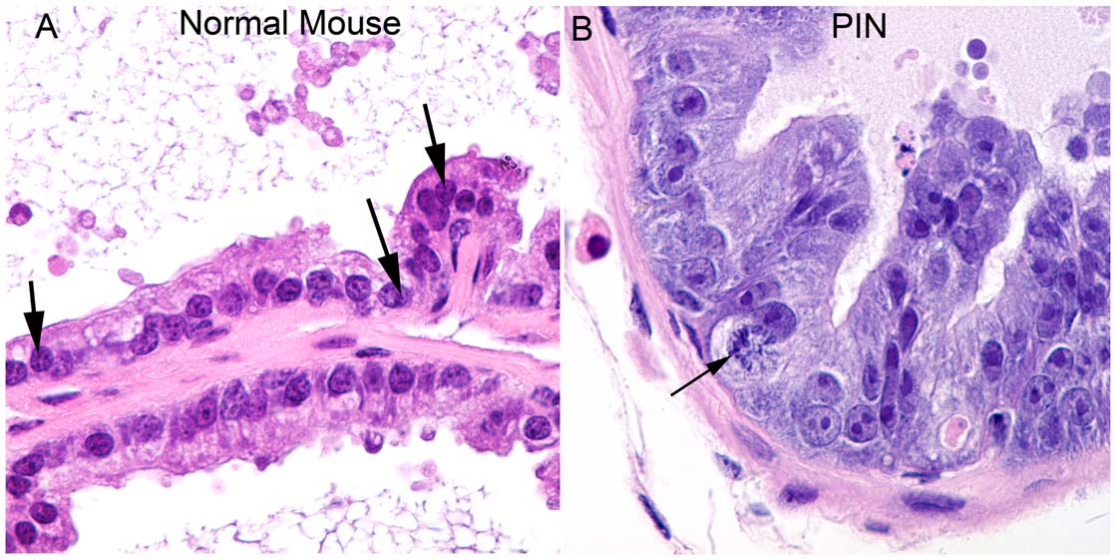
Morphological appearance of PIN lesions in the Lo-MYC mice in H&E stained tissue sections. This high power view shows the marked nucleolar enlargement as well as global changes in chromatin architecture including large-scale chromatin clearing that occurs in the mouse PIN cells as compared to the normal mouse prostate epithelial cells in an 8 week-old Lo-MYC mouse ventral prostate (original magnification 600×). Note that the mouse normal epithelial cells (A) show a relatively homogeneous chromatin distribution that is interrupted by a number of darkly staining punctate regions that are quite characteristic of mouse chromatin (arrows). This homogeneous chromatin distribution and the darkly staining regions are virtually abolished in the PIN cells (B) as a direct consequence of the MYC overexpression. The normal appearing epithelium contains only small nucleoli and much smaller nuclei than the PIN cells. Arrow in B indicates mitotic figure in PIN lesion.

**Figure 2 pone-0009427-g002:**
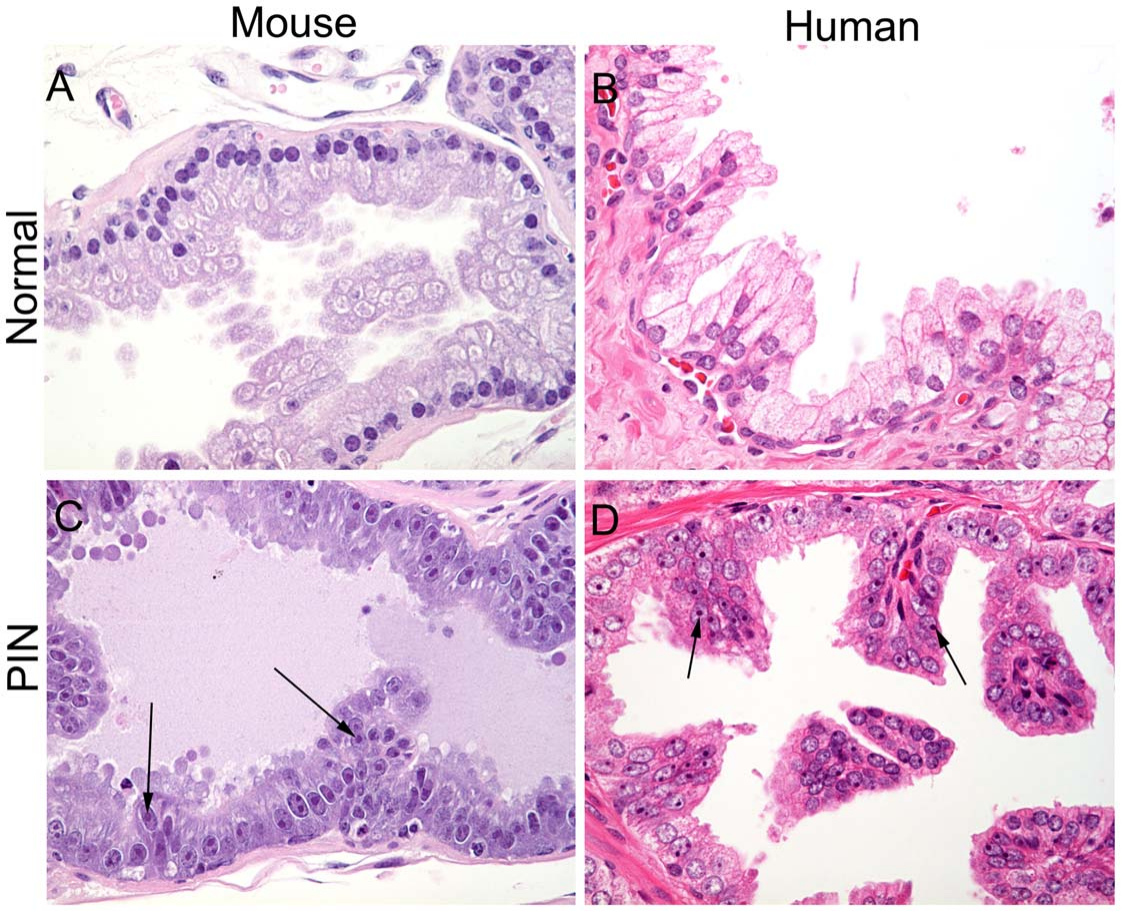
Morphological comparison of mouse and human normal appearing epithelium and PIN lesions. All images were taken at the same magnification and processed similarly to allow direct comparisons (400 ×). (A) Normal appearing mouse epithelium. (B) Normal appearing human prostate epithelium. (C) PIN lesion in a Lo-MYC mouse prostate. (D) PIN lesion in a human prostate. Arrows indicate enlarged nucleoli.

Since the ventral prostate was the most susceptible lobe in terms of the development of prostatic neoplasia in our study of Lo-MYC mice, it was chosen as the focus of most of our studies presented herein. The morphology of mouse PIN lesions was quite similar to that found in the human (**Figures 1** **–** **2**). The characteristic changes of PIN include cytoplasmic hyperchromasia, nuclear and nucleolar enlargement, and global changes in chromatin pattern. These global chromatin changes predominantly consist of changes in distribution such that in normal appearing cells the chromatin is nearly homogeneously distributed, but in PIN cells there are areas of chromatin clearing. This is accentuated somewhat in mouse PIN, since in normal mouse nuclei there are highly characteristic darkly staining punctate areas of chromatin that are distributed throughout the more homogeneously staining regions, and, these punctuate areas are largely absent in the mouse PIN cells (**Figures 1** **–** **2**). Human high grade PIN consists of a number of morphological variants. The most common variant is referred to as “tufting”, and an example of this is shown in **Figure 2**. Like human high grade PIN, the Lo-MYC PIN cells retained abundant amounts of cytoplasm and maintained cellular polarity, such that they retained a columnar appearance (like normal prostate luminal cell epithelium), with basally located nuclei and apically located cytoplasm (**Figures 1** **–** [Fig pone-0009427-g002] **3**). Also similar to most human high grade PIN lesions, the glandular architecture of the PIN lesions in Lo-MYC mice generally showed either a single cell layer, was pseudostratified, or contained what appeared to be multiple layers; albeit even in multilayered appearing PIN lesions the cells largely maintained their polarity (see **Figure 2** for direct comparison of mouse and human high grade PIN). In addition, the glands in mouse PIN maintained open lumens without cribriform formation or the development of multi-layered solid glandular structures. There was an increase in both mitotic figures (**Figure 1**) and cells with the morphological appearance of apoptosis in mouse PIN glands (most of which could be verified by IHC staining for cleaved caspase 3, not shown). As a comparison, we examined a number of mice from the Hi-MYC strain, and the histological features were similar, although the rate of progression to pre-invasive (see below) and invasive disease, as previously reported [1], was faster in Hi-MYC. Also, in Hi-MYC the lobe distribution of lesions is different and expanded [1] when compared to Lo-MYC. Since all PIN lesions showed marked nucleolar enlargement, similar to human high grade PIN, we propose to refer to these lesions as high grade PIN and we submit that Lo-MYC and Hi-MYC mice do not develop low grade PIN. As expected, the fraction of cells staining for Ki67, a measure of cellular proliferation, was markedly increased in the PIN lesions, as well as in other more advanced lesions described below (**Figure S1**).

**Figure 3:**
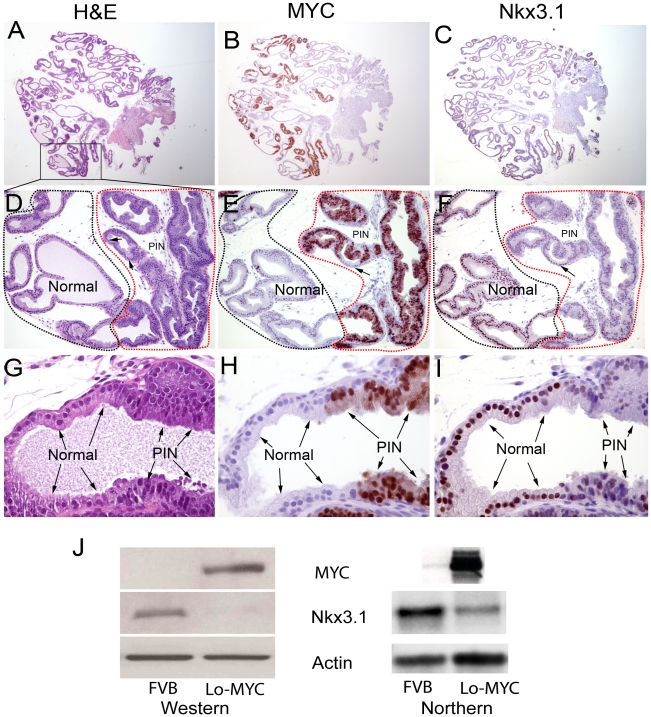
MYC overexpression corresponds precisely to morphological changes in luminal cells that are characteristic of PIN. (A–C). Low power (original magnification 20×) image of an entire lobe of a mouse ventral prostate at four weeks of age from a Lo-MYC mouse. Stains are indicated above images. Sections are not directly adjacent but are within ∼25–40 µM (of each other. Note the highly heterogeneous nature of the staining for MYC and Nkx3.1. (D–F). Medium power (100 ×) view of regions shown in the box in Figure 3A. Regions corresponding to normal appearing epithelium are circled in black, and regions corresponding to PIN lesions are circled in red. Note that regions of PIN are staining positively for MYC and the same regions are staining at a much reduced level for Nkx3.1. Also note small area in panel E that lacks staining for MYC, indicated by the arrow, which has strong positive staining for Nkx3.1 (indicated by the arrow in panel F). (G–I). Higher power image of region of transition between normal epithelium and PIN (the lowermost gland in panels D–F). Here it is clearly evident that cells corresponding to PIN cells, as shown in the H&E section (panel G) are the same cells that are staining strongly positive for MYC, and the same cells that have markedly reduced staining for Nkx3.1. (J) Western blot (left) showing reduced Nkx3.1 protein in a Lo-MYC mouse ventral prostate with PIN, as compared to an FVB wild-type mouse at the same age (six months). Northern blot (right) showing reduced *Nkx3.1* mRNA in a Lo-MYC mouse ventral prostate with PIN as compared to an FVB mouse at the same age (9 weeks). Top right shows *MYC* mRNA is highly elevated in another Lo-MYC mouse ventral prostate as compared to a matched FVB wild-type (9 weeks).

### Mapping the Onset and Dynamics of MYC Protein Expression and Its Relationship to Nuclear Architectural Alterations

We sought to determine precisely when MYC overexpression in the Lo-MYC mice occurs and to correlate this with the onset of morphological changes diagnostic of PIN. In the study by Ellwood-Yen et al., the authors suggested that overexpression of MYC was sufficient to cause early neoplastic transformation [1]. Western blot analysis at four weeks of age using the human-specific 9E10 antibody revealed increasing levels of MYC protein expression in Lo- MYC and Hi-MYC mice [1]. However, a precise correlation between the onset of MYC expression *in situ* and morphological transformation was not reported upon [1], most likely as a result of the poor performance of the 9E10 antibody (AM De Marzo, unpublished observations) and other anti-MYC antibodies in immunohistochemical assays. Therefore, until now it has not been clear whether MYC that was detectable on western blot in PIN lesions in Lo-MYC and Hi-MYC mice represented relatively low levels of expression in all prostate cells, or whether a subset of cells expressed MYC at relatively high levels. To circumvent this problem, we performed immunohistochemistry using a newer commercially available rabbit monoclonal antibody against MYC [31].

Using this antibody, detection of MYC protein was not seen in all of the nuclei of the luminal epithelial cells, but rather was heterogeneous and somewhat focal in expression, especially in the very young mice (e.g. less than 8 weeks of age) (**Figure 3**). Strikingly, MYC expression coincided precisely with the morphological changes characteristic of PIN (**Figure 3**). These findings of coincident staining of MYC and morphological transformation were identified in every mouse that we examined that showed PIN, including other prostate lobes in Lo-MYC mice and a number of Hi-MYC mice containing PIN lesions in various prostate lobes (more than 50 mice with PIN have been analyzed to date). While PIN lesions showed an increase in cells undergoing apoptosis (see above) we did not find increased levels of apoptosis, or cell proliferation, in regions of normal appearing prostatic epithelium in the Lo-MYC or Hi-MYC mice. Thus, it does not appear that induction of MYC overexpression in normal epithelial cells results in the induction of apoptosis. These findings suggest that accumulation of MYC protein is sufficient to transform prostate luminal epithelial cells into PIN cells *in vivo* without a concomitant need for additional alterations required to suppress apoptosis. Wild type adult mice were often completely negative for MYC staining, but some mice contained weak expression of MYC that was predominantly found within the nuclei in the luminal compartment and that we subsequently validated as authentic mouse Myc by western blotting (data not shown).

### PIN Develops Exclusively in Prostatic Luminal Cells in Lo-MYC and Hi-MYC Mice

**Figure 4:**
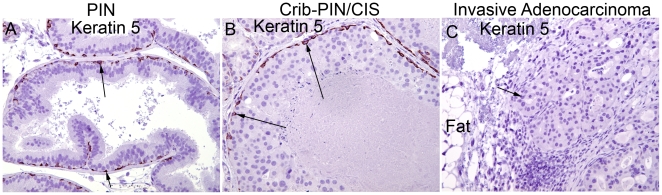
Neoplastic appearing PIN and cribriform PIN/CIS cells and adenocarcinoma cells are negative for basal cell specific Keratin 5 staining (original magnification 200×). (A) A mouse PIN lesion from a Lo-MYC mouse showing that neoplastic appearing cells are negative for Keratin 5 and that there is a largely intact layer of benign appearing flattened basal cells. (B) A similar basal cell layer is highlighted by staining positively for Keratin 5 in a cribriform PIN/CIS lesion. (C) A frankly invasive adenocarcinoma lesion in a Lo-MYC mouse, showing invasion into the peri-prostatic fatty tissue, that is completely devoid of basal cells or any tumor cell Keratin 5 staining. Arrow indicates invasive adenocarcinoma gland.

A recent study indicated that in mouse tumors that develop after prostatic-specific deletion of *Pten* (driven by ARR_2_-*Probasin*-Cre), the PIN cells appear to originate in the proximal regions of the ducts [64]. Interestingly, the cells implicated were basal-like cells with characteristic mouse prostate stem cell markers (e.g. Keratin 8-, Keratin 5+, p63+, Sca 1+, CD49f+), or transiently proliferating cells with a phenotype between basal and luminal cells (p63-, Keratin5+, Keratin 8+)—luminal cells did not appear to be the target of transformation [64]. In our studies of Lo-MYC and Hi-MYC mice, morphological appearances indicate strongly that only luminal cells, and not basal cells, are positive for high levels of MYC staining and show morphological features of PIN. Further, we found no proximal-distal predilection to the development of PIN lesions. To more definitively determine whether basal cells were potential targets of transformation in these mice, we stained mouse prostates containing PIN from Lo-MYC mice with an anti-Keratin 5 antibody. **Figure 4** shows that cells that were positive for Keratin 5 were localized exclusively within the basal compartment along the basement membrane and did not show features of enlargement or a morphological appearance of transformation. In addition, we performed double-label immunofluorescence in a number of Lo-MYC and Hi-MYC mice with antibodies against p63 (another basal cell marker) and MYC, and we found that in PIN lesions only cells devoid of p63 nuclear staining were strongly positive for MYC (data not shown). These results indicate that only luminal-like cells, without Keratin 5 or p63, are targets of transformation in Lo-MYC and Hi-MYC mice.

### Inverse Relationship between C-MYC and Nkx3.1 in PIN Lesions

In the prior study by Ellwood-Yen et al., Hi-MYC mice showed decreased Nkx3.1 mRNA and protein expression, with some variable reduction in PIN lesions, and virtual complete reduction in invasive adenocarcinoma lesions [1]. However, without MYC IHC staining, the relation between the onset of MYC expression and Nkx3.1 reduction could not be determined. Further, Ellwood-Yen et al suggested that Nkx3.1 loss was a distinct event from the onset of MYC expression such that its loss together with MYC overexpression may be a critical cooperating event in mouse PIN to adenocarcinoma transition [1]. In the present study using a different anti-Nkx3.1 antibody raised against mouse Nkx3.1[61], while very intense nuclear staining for Nkx3.1 was seen in virtually all normal luminal cells in the mouse prostate (in all lobes), its expression was dramatically decreased in PIN in all cases in Lo-MYC and Hi-MYC mice examined (**Figures 3**). There appeared to be a cell-by-cell correspondence between increased MYC and decreased Nkx3.1 protein in PIN lesions. Decreased Nkx3.1 protein in Lo-MYC mice was also verified by western blotting of ventral prostate lysates (**Figure 3J**). Thus, unlike the findings by Ellwood-Yen et al., and more recently Song et al.,[65] that identified marked reductions of Nkx3.1 only in invasive adenocarcinoma in MYC overexpressing mice [1], we identified such reductions in PIN. In fact, as compared to mouse PIN lesions we see increased Nkx3.1 in early pre-invasive and invasive lesions (see below). That reduced levels of Nkx3.1 itself are not responsible for the morphological changes diagnostic of high grade PIN is evidenced by the findings that *Nkx3.1* ^(−/−)^ knockout mice [52], [53], [54], or mice with targeted disruption of *Nkx3.1* alleles in adulthood [66], show only very subtle changes such as hyperplasia and mild dysplasia (PIN-like), and even these are clearly much less pronounced that that seen in the Lo-MYC and Hi-MYC mice.

While the mechanism(s) by which MYC regulates Nkx3.1 protein in the mouse prostate is not clear, Ellwood-Yen et al. showed that mouse *Nkx3.1* mRNA was reduced by MYC [1], suggesting that the regulation may at least in part be at the level of mRNA. To determine whether reduced Nkx3.1 protein was related to reduced *Nkx3.1* mRNA levels in the ventral prostates of Lo-MYC mice, we performed Northern blot experiments. **Figure 3J** shows a clear reduction in *Nkx3.1* mRNA in Lo-MYC at 9 weeks of age, a time in which the majority of luminal epithelial cells appear to be PIN cells (see below). Further, in preliminary experiments in human prostate cancer cells that express NKX3.1 (LNCaP cells), knockdown of MYC by siRNA resulted in an increase in *NKX3.1* mRNA and protein, showing that MYC can indeed regulate *NKX3.1* at the mRNA level (CM Koh, AM De Marzo, manuscript in process).

### Generation of Super-Lo-MYC Mice

A previously reported mouse strain in which mouse *Myc* was driven by the rat C(3)1 promoter [32], which appears to be weaker than the *probasin* promoter, found that low grade PIN lesions developed with Myc overexpression, albeit these lesions did not progress to invasive adenocarcinoma. Unfortunately this strain of mice was lost by the time of the initial publication [32] so that we cannot directly compare the levels of MYC in these mice to those of the Lo-MYC mice.

**Figure 5:**
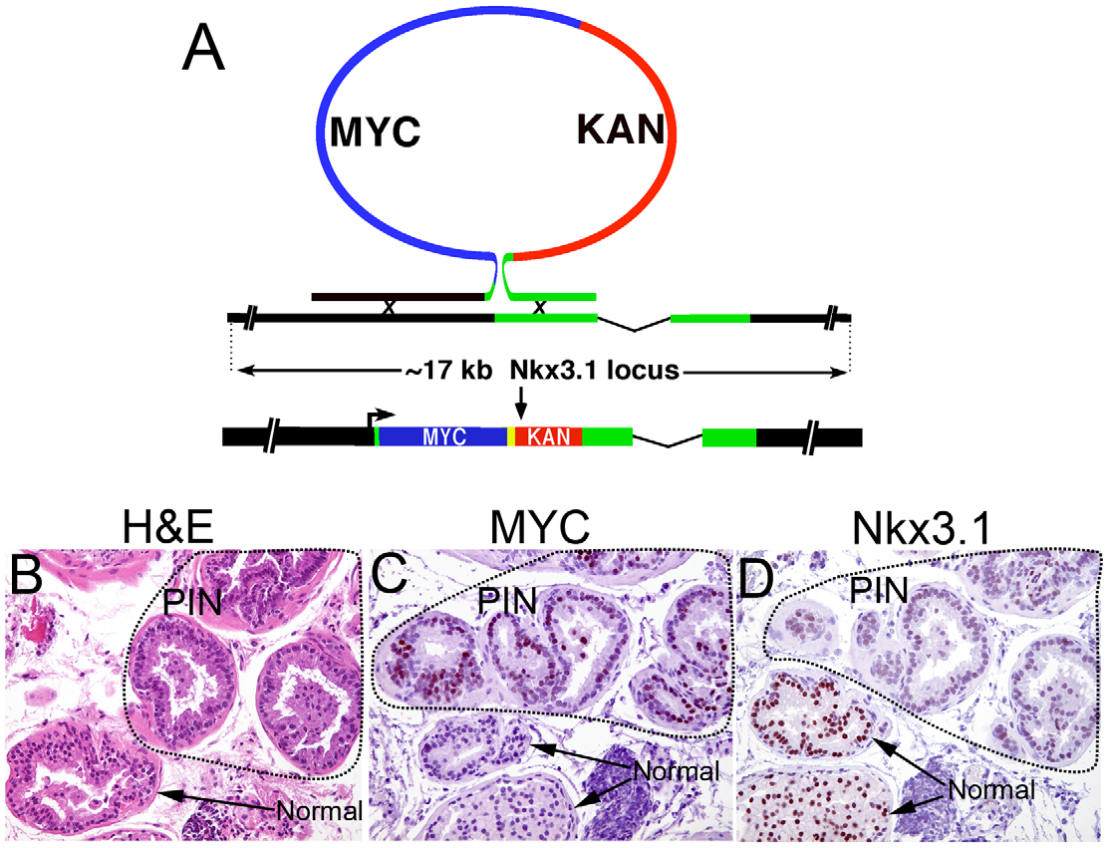
Generation and characterization of the “Super Lo-MYC” mouse model. (A) Schematic representation of the recombineering approach used to generate the *Nkx3.1-MYC* transgene. A MYC/KAN cassette flanked by *Nkx3.1* homologous arms was recombined in bacteria with a vector containing ∼17 kb of the *Nkx3.1* genomic locus. In the final transgene construct, the *MYC*-encoding mRNA initiates at the *Nkx3.1* transcriptional start site and the SV40 early poly(A) signal (yellow box) mediates polyadenylation. (B–D) PIN lesion developing in a Super Lo-MYC mouse. All magnifications are 100×. (B) H&E section in which PIN lesion is circled. (C) MYC IHC staining showing that a large fraction of cells in the PIN lesion are staining weakly to moderately positive. (D) NKX3.1 is reduced in the same PIN appearing cells that are positive for MYC staining.

Therefore, we sought to address this issue of whether low levels of MYC overexpression could cause transformation in mouse prostatic epithelial cells in a new set of experiments. In order to address whether forced overexpression of MYC at levels much lower than those seen in Lo-MYC and Hi-MYC mice can also result in PIN and/or adenocarcinoma, we generated a new mouse strain that overexpresses human MYC in the prostate. For this we used an *Nkx3.1* cis-acting region, which has been previously shown to result in prostate epithelial cell specific expression of a reporter gene [61] to target human MYC expression to the mouse prostate epithelium. Thirteen of 81 founder generation mice carried the transgene, and offspring were successfully derived from 10. Analyses of transgene expression by Northern blot and immunohistochemistry revealed that only a single line expressed MYC in the prostate. Since MYC may downregulate *Nkx3.1* at the level of transcription (see above), it is possible that elevated MYC itself may be responsible for down-regulating *Nkx3.1* expression. Thus, it appears that only relatively low levels of MYC can be driven by this transgene. The levels of MYC in these newly generated mice appeared substantially lower than the levels seen in the Lo-MYC or Hi-MYC mice when analyzed by IHC (**Figure 5**). Nevertheless, in this mouse, which we designate “Super-Lo-MYC”, we do see changes consistent with foci of low grade PIN (**Figure 5**). Importantly, while the degree of nuclear and nucleolar enlargement is less that that seen with Lo or Hi-MYC mice, these changes in Super-Lo-MYC mice localize precisely to the cells that overexpress MYC protein by IHC (**Figure 5**). These PIN lesions also showed an increase in Ki67 staining fraction, and an increase in apoptosis, albeit not nearly as pronounced in Lo-MYC or Hi-MYC (not shown). These results appear similar to the previous findings using the rat C(3)1 promoter [32], and suggest that even moderate overexpression of MYC in the prostate results in cells with the appearance of PIN (albeit low grade), and not in apoptosis or cell cycle arrest.

### Characterization of Cribriform PIN/Carcinoma *In Situ* as Pre-Invasive Lesions

Most mouse models of human prostate cancer that have been reported to date appear to progress through a phase in which the morphological appearance of PIN lesions is distinctly different than most human PIN lesions. In particular, unlike human PIN lesions, the neoplastic lesions in these mouse models often fill or nearly fill the lumens of the glands, as either solid sheets, as cribriform structures, or as tufting or papillary structures [67], [68]. Also, while some models show recognizable neoplastic lesions (e.g. PIN) prior to the development of these more solid glandular structures, the degree of nuclear atypia (which does not appear to approach the degree we observe in models based on MYC overexpression), generally does not increase significantly until the neoplastic lesions have filled or nearly filled the lumens of the glands.

**Figure 6:**
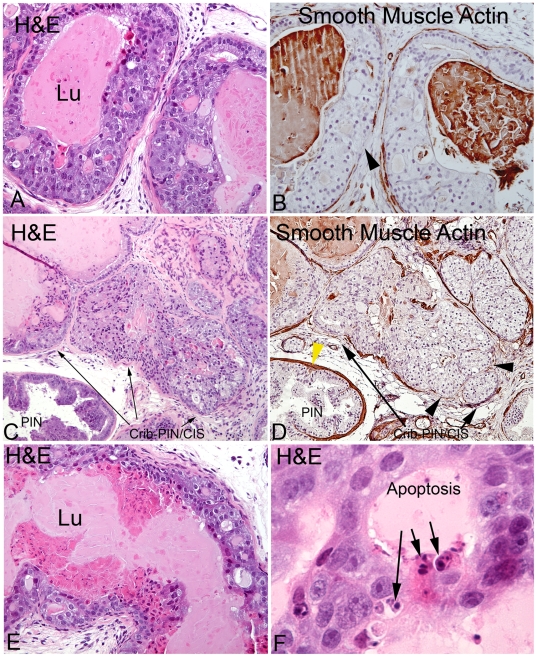
Characterization of cribriform PIN/CIS lesions in the Lo-MYC mouse model. (A) H&E section of an early cribriform PIN/CIS lesion, without microinvasion, showing multilayering of cells partially filling the lumen. Note the loss of columnar cell polarity and the variable decrease in nucleolar size as compared to PIN lesions shown in Figures 1–3 (original magnification 200×). (B) Smooth muscle actin staining showing a highly attenuated smooth muscle cell layer in the same lesion from an adjacent section (original magnification 200×). (C) H&E stained section of a more advanced cribriform PIN/CIS lesion that shows early development of microinvasion, which is better shown in panel (D) by smooth muscle actin staining (original magnification 100×). Black arrowheads showed regions of complete smooth muscle loss. Yellow arrowhead shows intact smooth muscle layer around a PIN gland (C–D, original magnification 100×). (E) H&E section of another cribriform PIN/CIS lesion showing apoptosis and necrosis towards the lumen (original magnification 200×). (F) Higher magnification view (600×) showing morphological evidence of apoptosis in a different cribriform PIN/CIS lesion.

In distinct contrast, in Lo-MYC and Hi-MYC mice, as indicated above there is marked nuclear atypia at the earliest recognizable stages, as in human PIN. Nevertheless, the PIN lesions in these mice do evolve into cribriform lesions as an apparent progression event (**Figures 6** **and** **7**). In addition to multi-layering of cells, these changes in Lo-MYC were accompanied by very distinctive cytological features which consist of a variable decrease in cytoplasmic hyperchromasia, loss of polarity and increased nuclear size and shape variability (e.g. pleomorphism) (**Figures 6** **–** **7**). The loss in polarity was characterized by a loss in basally located nuclei within the PIN cells such that the cells exhibited much more variation in the location of nuclei within them. These changes were also accompanied by an increase in the variation of nucleolar size and shape. Overall, there was a reduction in nuclear and nucleolar size as compared with high grade PIN lesions, although these atypical nuclear characteristics were still much more prominent than seen in wild type mice (**Figures 6** **–** **7**).

**Figure 7:**
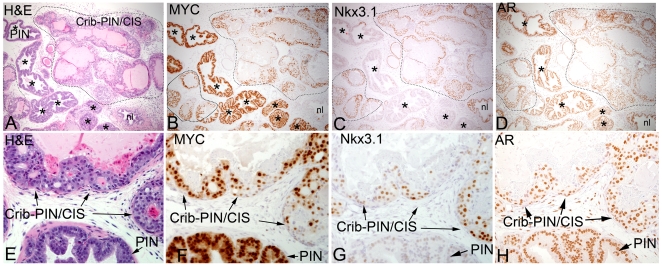
MYC levels are reduced and Nkx3.1 protein is markedly increased in cribriform PIN/CIS lesions. (A–D) Low power view (original magnification 40×) of the ventral prostate from a Lo-MYC MYC mouse at 1 year of age that contains residual PIN glands (*) and cribriform PIN/CIS (circled in black dotted line). (E–H) A higher power view (original magnification 200×) of a different mouse showing both PIN and cribriform PIN/CIS in the same microscopic field. Immunostains as indicated show a moderate reduction in MYC staining overall and a marked increase in Nkx3.1 in cribriform PIN/CIS areas. AR expression is retained in both PIN and cribriform PIN/CIS.

These morphological alterations in the cribriform PIN/CIS lesions were also accompanied by molecular alterations. Interestingly, MYC expression was somewhat decreased and staining for Nkx3.1 was increased in many of these cells (**Figure 7**). Also, as the cells piled up further, there was a marked increase in apoptosis and frank necrosis towards the lumens (**Figure 6E–F**). Necrosis has been identified in other genetically engineered mouse models of human prostate cancer, and when it occurs, the authors refer to the lesions as PIN IV [67].

**Figure 8:**
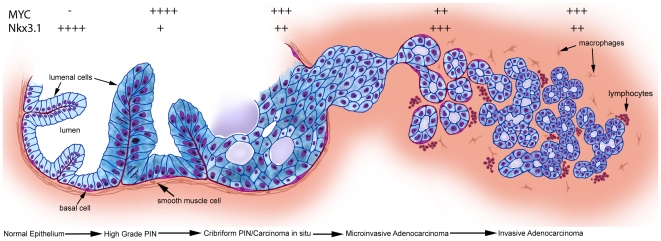
New model of progression from normal to PIN to cribriform PIN/CIS to microinvasive adenocarcinoma in Lo-MYC and Hi-MYC mice. Relative levels of nuclear staining for Nkx3.1 and MYC are indicated. In normal epithelium, MYC levels are generally undetectable (−) although low levels can be seen. Conversely, very high levels (++++) of Nkx3.1 are present in normal epithelium. In high grade pin lesions, MYC levels are markedly elevated (++++), and Nkx3.1 levels are markedly reduced (+) as compared to normal epithelium. In cribriform PIN/in situ carcinoma lesions, MYC levels are reduced somewhat compared to high grade PIN (+++), although still markedly above levels in normal epithelium. Here, Nkx3.1 levels are elevated (++) as compared to high grade PIN, although they are still somewhat reduced compared to normal epithelium. As invasive tumors become somewhat larger, MYC levels increase again (+++), although they are not quite as elevated and are more variable than in the high grade PIN lesions. Nkx3.1 levels (++) are also more variable here.

That the cribriform PIN/CIS lesions are the precursors to invasive adenocarcinoma was evidenced by the finding that in all cases in which we identified microinvasive adenocarcinomas, there appeared to be transitions into structures that were in the process of microinvasion. As shown by reductions in smooth muscle actin staining around the glands [67], the microinvasive lesions appear to emanate from these morphologically transformed cribriform, or partially cribriform lesions (**Figure 6** **–** **7**). The degree of nuclear atypia decreased even further in the microinvasive lesions. We interpret these findings as a kind of “paradoxical differentiation” occurring just prior to and at the point of invasion. Interestingly, in most of the Cribriform-PIN/CIS lesions, there was a maintenance of at least some basal cells, which were identified by staining for Keratin 5 (**Figure 4B**) and p63 (not shown), despite the often near complete loss of smooth muscle actin staining (**Figures 6B and 6D**). Only in a few of the more advanced lesions in Lo-MYC mice, which in some cases showed invasion into the surrounding prostatic fat tissue (**Figure 4C**), was there evidence for complete loss of basal cells, indicative of unequivocal invasive adenocarcinoma. Additionally, regions of early invasion showed a mild to moderate lymphocytic inflammatory cell infiltrate around the tumor cells (**Figure 4C**). IHC staining (CD3, CD4, CD8, CD20, F4/80) indicated that most of these inflammatory cells are T cells, with some B cells and a moderate number of macrophages as well (not shown). **Figure 8** shows our current progression model for these lesions.

**Figure 9:**
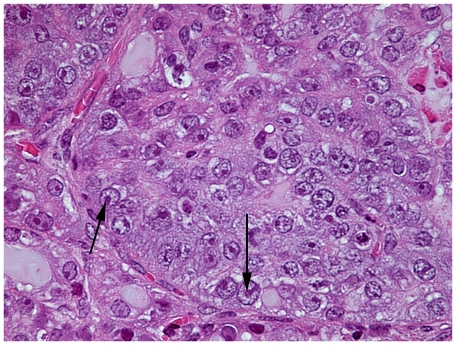
Nuclear atypia resumes in larger adenocarcinoma lesions. High power view (original magnification 400×) of an H&E stained section of invasive adenocarcinoma lesion in Lo-MYC mouse showing cells with large nucleoli, similar to that seen in PIN lesions. These lesions stain nearly as strongly for MYC as PIN lesions, and there is also fairly high levels of Nkx3.1 protein (e.g. much higher than PIN and similar to cribriform PIN/CIS).

Since Androgen Receptor (AR) staining did not appreciably change during the preinvasive and microinvasive processes (**Figure 7**), it appears that alterations in AR levels cannot be responsible for dynamic changes in MYC, Nkx3.1, or the morphological changes. Although a number of Lo-MYC mice were followed for up to 2 years of age (**Table 1**), most lesions in mice that were 1 year or older showed numerous foci of cribriform PIN/CIS, often with apparent microinvasion—no metastatic lesions were identified. In the few examples in which the invasive lesions appeared to grow larger in Lo-MYC mice, the MYC expression increased and the Nkx3.1 staining was reduced, but overall the Nkx3.1 staining was quite variable and much higher than in the PIN lesions (not shown). The degree of nuclear atypia (nucleolar enlargement and pleomorphism) increased in these larger lesions as well (**Figure 9**).

**Table 1:** The phenotype of ventral prostate (VP) and lateral prostate (LP) in Lo-MYC mice and wild type mice.

Age	VP in Lo-MYC	LP in Lo-MYC	Wild type
2W	4/4 benign		4/4 benign
4W	6/6 PIN	1/6 PIN5/6 benign	4/4 benign
8W	1/8 PIN with cribriform PIN/CIS7/8 PIN	5/8 PIN3/8 benign	3/3 benign
12W	4/9 PIN with cribriform PIN/CIS5/9 PIN	3/8 PIN with cribriform PIN/CIS4/8 PIN1/8 benign	4/4 benign
16W	3/3 PIN	2/3 PIN1/3 benign	3/3 benign
5M	4/10 PIN with cribriform PIN/CIS6/10 PIN	10/10 PIN	3/3 benign
7M	6/16 PIN with cribriform PIN/CIS10/16 PIN	1/16 PIN with cribriform PIN/CIS9/16 PIN6/16 benign	7/7 benign
10M	2/3 PIN with cribriform PIN/CIS1/3 PIN	2/3 PIN1/3 benign	3/3 benign
12M	7/7 cribriform PIN/CIS with micro invasive adenocarcinoma	5/7 cribriform PIN/CIS with micro invasive adenocarcinoma1/7 PIN1/7 benign	4/4 benign
15M	4/4 cribriform PIN/CIS with micro invasive adenocarcinoma	1/3 invasive adenocarcinoma1/3 PIN with cribriform PIN/CIS1/3 benign	3/3 benign
18M	3/3 cribriform PIN/CIS with micro invasive adenocarcinoma	2/3 cribriform PIN/CIS with micro invasive adenocarcinoma1/3 PIN	3/3 benign
21M	3/3 cribriform PIN/CIS with micro invasive adenocarcinoma	1/3 cribriform PIN/CIS with micro invasive adenocarcinoma1/3 PIN1/3 benign	3/3 benign
24M	10/10 cribriform PIN/CIS with micro invasive adenocarcinoma	7/10 cribriform PIN/CIS with micro invasive adenocarcinoma2/10 PIN1/10 benign	4/4 benign

In all cases, “invasive adenocarcinoma” indicates microinvasion into stroma as described in the text.

In Hi-MYC mice, the lesions also progressed from high grade PIN to cribriform PIN/CIS prior to the development of frank invasive adenocarcinoma. Also, in Hi-MYC mice the reduction in MYC protein seen in the cribriform PIN/CIS lesions and early invasive lesions was present, but was not nearly so pronounced as in the Lo-MYC mice (data not shown).

### Frequency and Lobe Predilection of PIN and Early Carcinoma Development

The overall frequency and lobe predilection for the development of PIN and early invasive adenocarcinoma lesions in Lo-MYC were similar to that reported by Ellwood-Yen et al. (Table 1) [1]. All mice developed PIN in the ventral prostate by 4 weeks (**Figures 1** **–** [Fig pone-0009427-g002] **3**). By 8 weeks of age most ventral prostates contained PIN that occupied between 50 and 100% of the lobe. By one year of age (52 weeks), all mice developed microinvasive adenocarcinoma lesions, consisting of lesions apparently in transition to true invasion as in **Figures 4** **,** **6** **–** **7** and those that are frankly invasive (**Figure 4C**). Between 4 weeks and 52 weeks, variable amounts of PIN, and cribriform PIN/CIS with some apparent microinvasion predominated. In the lateral prostate, 5 out of 8 cases showed PIN at 8 weeks of age without invasion. By 52 weeks of age, 5 out of 7 mice developed microinvasive adenocarcinoma that also appeared to be derived from cribriform PIN/CIS. In general, the area of PIN, cribriform PIN/CIS, and microinvasive adenocarcinoma lesions in the lateral prostate was markedly less than that in the ventral prostate at the same age. The anterior prostate did not show any neoplastic lesions, except that a single mouse developed PIN at 18 months of age. The dorsal lobe did not show any neoplastic lesions.

## Discussion

We used the Lo-MYC mouse model to assess the onset and dynamics of MYC protein accumulation within epithelial cells during the development of PIN and the progression to localized invasive prostatic adenocarcinoma. We show that the onset of MYC protein overexpression coincides precisely with the morphological transformation of mouse prostate luminal epithelial cells into PIN cells—an appearance that is highly similar to human high grade PIN. These changes include striking nucleolar size expansion, large-scale chromatin redistribution, cytoplasmic hyperchromasia, an increase in cell density, a marked increase in proliferative fraction, and an increase in apoptosis. These results support the hypothesis that overexpression of MYC, in the absence of additional changes in other genes or pathways such as those required to suppress MYC-induced apoptosis [1], [33], is sufficient to directly transform prostatic luminal epithelial cells into PIN cells [1], [32], [33]. Since MYC overexpression occurs within luminal epithelial cells in PIN lesions in human cells [31], the data are also consistent with the hypothesis that epithelial cells within the luminal compartment are the target cells for transformation in the human prostate [19], [20], [22], [25], [31], [69], [70]. Whether any differentiated epithelial cell within the luminal compartment can be the target of MYC-induced transformation, or whether highly specialized luminal cells such as CARNs[25] are the target, will require further experimentation. In the current study, we also found an inverse relation between the expression of MYC and the phenotypic transcription factor/tumor suppressor, Nkx3.1 in PIN lesions. Finally, our detailed examination of prostate morphology in Lo-MYC (and Hi-MYC) mice uncovered an additional intermediate step (cribriform PIN/CIS) in progression from mouse PIN to invasive carcinoma. All of the morphological features and changes that occur in Lo-MYC were also identified in Hi-MYC, albeit with accelerated kinetics in Hi-MYC.

### Dynamic Expression of MYC during Disease Progression

In Lo-MYC mice, the levels of MYC in the pre-invasive cribriform PIN/CIS lesions and in early adenocarcinoma lesions were decreased somewhat as compared to their precursor high grade PIN lesions. Within the cribriform PIN/CIS lesions, the largest reductions in staining were in cells towards the lumens. These peri-luminal regions also showed an increase in both apoptosis and necrosis. It is well recognized that cells residing towards the lumens in multilayered cribriform and/or solid intra-glandular neoplastic lesions have decreased access to blood vessels and, therefore, decreased access to both nutrients and oxygen. Since high levels of MYC result in high nutrient demands on the rapidly proliferating neoplastic cells, we postulate that these cells are either unable to survive (thus undergoing apoptosis or necrosis), or, they have found a way to reduce MYC levels, which in turn would reduce their level of nutrient and oxygen use. Since AR levels did not appear to change in these lesions, the reductions in MYC cannot be explained by reductions in AR. Although Hif-1α, which is stabilized and overexpressed in regions of hypoxia, has been shown to repress MYC levels in other systems [71], we did not find an increase in levels of Hif-1α staining either toward the lumens of cribriform neoplastic glands, nor at the invasive front of budding early adenocarcinoma lesions (T. Iwata, AM De Marzo, unpublished results). When we examined hypoxia levels more directly using pimonidazole labeling, we did find a small increase in hypoxia-related staining in these inner regions near the necrotic/apoptotic centers of the cribriform lesions, but we did not see the same increase at the invasive fronts in the early invasive lesions (T. Iwata, AM De Marzo, unpublished results). Thus, it is likely that reductions in nutrient supplies, rather than hypoxia, may be a more important feature driving this phenotypic switch. It is likely that the morphological appearance of the PIN cells, which changed precisely at this time of cribriform PIN/CIS development (the cytoplasmic hyperchromasia was reduced and cellular polarity was lost), also reflect an underlying nutrient and/or hypoxic stress. The similar morphological and phenotypic features of the cribriform PIN/CIS cells, and the early invasive epithelial cells, suggest that the same decrease in nutrient supply may be experienced by cells that have just invaded the stroma. This is also not unexpected since it is likely that early invasive lesions would not possess an elaborate and highly functional vascular network. The reduction in nucleolar size, the decrease in cytoplasmic hyperchromasia, the increase in cytoplasm, and increase in Nkx3.1 in the preinvasive and early invasive lesions suggest that these cells are differentiating/maturing, albeit aberrantly. Similar characteristics are well known to occur in some human preinvasive lesions as they begin to invade the stroma (e.g. squamous cell carcinoma in many body sites, and urothelial carcinoma) [72], and, McNeal et al. proposed similar findings for human prostate cancer early invasion [73]. The recovery of MYC protein and more prominent nuclear atypia (e.g. markedly enlarged nucleoli) were observed upon enlargement of the invasive tumors, as angiogenesis increases [1]. Although the extent was less, these changes, which are suggestive of paradoxical differentiation, and slowed growth, also occurred in Hi-MYC mice.

### Dynamic Expression of Nkx3.1 during Disease Progression

The prevailing model of NKX3.1 expression in human prostate cancer suggests that while the protein may decrease in PIN lesions, it is much more commonly decreased in invasive adenocarcinomas, and nearly completely lost in most, if not all, metastatic prostate adenocarcinomas [52], [59], [74]. The aspects of this model related to primary cancer lesions are generally supported by Ellwood-Yen et al. who found in Hi-MYC mice that there was a variable decrease in expression of Nkx3.1 in PIN lesions, and that Nkx3.1 was virtually completely lost in invasive adenocarcinomas [1]. In addition, Song et al. recently reported similar results [65]. However, several observations from our group regarding NKX3.1 differ from this prevailing view. First, in a previous report, while reductions of NKX3.1 protein occurred in PIN lesions and some adenocarcinomas, the reductions were relatively minor and virtually all invasive adenocarcinomas retained significant levels of NKX3.1 protein [57]. These results are similar to those reported by Korkmaz et al. [75]. More recently we have found that the majority of very high grade (Gleason score 8–10) localized prostate cancers [76], and even almost all metastatic lesions from hormone naïve patients (B Gurel, C Bieberich, AM De Marzo, manuscript in revision), retain high levels of expression of NKX3.1 protein. In the present study we found that, as compared to high grade PIN, the staining for Nkx3.1 protein actually increased substantially in pre-invasive cribriform PIN/CIS lesions and in early invasive adenocarcinomas, and these levels correlated inversely with levels of MYC expression. These results indicate the Nkx3.1 may be dynamically regulated during progression of this disease. Interestingly, Nkx3.1 levels are high in budding epithelial cells during prostate development [61], [74], and mice with targeted disruption of Nkx3.1 show reduced ductal branching morphogenesis in all prostate lobes [74]. It is possible, therefore, that Nkx3.1 expression in invasive prostatic acini in MYC-driven mouse prostate cancers may represent a recapitulation or caricature of the process of stromal invasion/branching morphogenesis in development, and, that Nkx3.1 may facilitate this process.

Lei et al, found that in animals that were subjected to conditional deletion of *Pten* in prostate epithelium, Nkx3.1 levels were reduced [77], indicating that wildtype Pten levels are required to maintain normal Nkx3.1 protein levels. Further, Lei et al. found that forced restoration of Nkx3.1 expression in *Pten* null epithelium led to decreased cell proliferation, increased cell death, and prevention of tumor initiation [77]. They further showed that Nkx3.1 was required to engage the p53 pathway, indicating that reduced Nkx3.1 expression can itself abrogate p53 signaling. These findings raise the interesting possibility that the reduction in Nkx3.1 seen upon the induction of MYC in the mouse prostate prevents the induction of p53 induced apoptosis, thus facilitating MYC's ability to transform these cells. Additional studies in which Nkx3.1 expression is kept at high levels during induction of MYC in prostate epithelium will be required to address this question further.

We do not know precisely how MYC is regulating Nkx 3.1 protein expression, although using gene expression array analysis Ellwood-Yen et al. described a decrease in *Nkx3.1* mRNA levels in Hi-MYC mice[1]. Also, we found a decrease in *Nkx3.1* mRNA in Lo-MYC mice and an increase in *Nkx3.1* mRNA levels in human prostate cancer cells (LNCaP) after siRNA induced MYC knockdown (C. Koh, AM De Marzo, unpublished observations). Although further work is needed to determine whether this effect is directly transcriptional, or post-transcriptional, these combined results indicates that MYC regulates *Nkx3.1* at the mRNA level.

In the somewhat larger invasive tumors, many cells have both high levels of MYC and high levels of Nkx3.1, which suggests that the tight regulation of Nkx3.1 by MYC in the PIN lesions appears to be lost to some degree in the invasive carcinoma lesions. That the regulation of Nkx3.1 levels is complex is evidenced by the fact that while Nkx3.1 levels are androgen regulated in adult prostate epithelial cells, these levels are not under androgen regulation during prostate development[59]. Also, Nkx3.1 is expressed in a number of other cell lineages during development, including somites, and these lineages are not under androgen regulation. In terms of transcriptional regulation of mouse *Nkx3.1*, a 5-kb fragment downstream of the *Nkx3.1* coding region was shown to contain elements that support expression in the prostate and bulbourethral glands, whereas an upstream fragment was found to contain elements that direct expression in somites and testes [61].

Compared to Lo-MYC mice, there was less of a reduction in MYC in the cribriform PIN/CIS and early invasive lesions in Hi-MYC mice. Yet, as in Lo-MYC, the levels of Nkx3.1 in these lesions from Hi-MYC were much higher than in PIN lesions. Taken together with the co-expression of high levels of MYC and Nkx3.1 in the more advanced invasive carcinomas from Lo-MYC mice, these results suggest that the large effects on downregulation of Nkx3.1 by MYC only occurs in PIN lesions, with less of an effect in pre-invasive and invasive lesions. This contention is also supported by the fact that in human high grade PIN, while there is reduction in the intensity of staining for NKX3.1 protein in most cases, most of these same cases did not show loss of chromosome 8p [57]. NKX3.1 protein levels, but not mRNA levels, have also been shown to be markedly decreased in human focal prostate atrophy (which is often associated with inflammation and designated as PIA) [57], [78], lesions that do not show clonal loss of chromosome 8p[57]. Consistent with this, the inflammatory cytokines tumor necrosis factor (TNF)-alpha and interleukin-1β have been shown to accelerate NKX3.1 protein loss by inducing rapid ubiquitination and proteasomal degradation [79]. Further, Li et al, have shown that NKX3.1 protein levels can be regulated by phosphorylation mediated by Protein Kinase CK2, which itself is under complex regulation [80]. Taken together, it is clear that the regulation of *NKX3.1* mRNA and protein levels is highly complex, and additional studies will be required to more fully determine in detail the molecular mechanisms of control of NKX3.1 levels in different human and mouse prostate lesions.

### Are Other Genetic or Epigenetic Changes Needed to Transform Prostate Epithelial Cells in Lo-MYC and Hi MYC Mice?

The coincident occurrence of nuclear atypia and MYC transgene expression strongly suggests that MYC accumulation alone is sufficient to transform prostate epithelial cells into PIN cells. It is likely that the reason that transgene expression does not occur throughout the entire prostate epithelium, as might be expected of the endogenous *probasin* promoter, is a result of the well-known fact that the site of integration can affect the pattern of expression in given transgenic lines[81]. In fact, quite variable expression of transgenes driven by the minimal rat *probasin* promoter, as well as larger promoter fragments, have been reported upon previously [82]. Nevertheless, one cannot rule out the possibility that there are other genes whose activity is responsible for repressing *MYC* transgene transcription, the accumulation of *MYC* mRNA, or the accumulation of MYC protein, whose function must be altered in order for MYC protein to ultimately accumulate in Lo-MYC and Hi-MYC mice (or in Super-Lo-MYC mice). While the need for a secondary collaborative event such as this is unlikely because of the very rapid onset of the development of PIN in both Lo-MYC and Hi-MYC mice, until an inducible system is developed in which the transgene can be expressed at will in all target cells, this question will remain somewhat open. Whether another cooperating event is required in order to allow the accumulation of MYC protein or not, it is still evident from our results that the accumulation of MYC protein alone, once it occurs, is sufficient to directly transform prostate epithelial luminal cells into PIN cells.

Somewhat contrary to these results, Kim et al. recently produced a different MYC-based mouse model in which MYC was activated by removing a floxed “STOP” sequence (present in the *Z-MYC* mice) using Cre recombinase driven by the Probasin promoter (present in the *Pb-Cre4* mice) [83]. In these compound transgenic *Pb-Cre4;Z-MYC* mice, while some mice overexpessing MYC showed evidence of PIN, there were a number of mice that apparently overexpressed MYC in the prostate epithelium that did not show PIN. Further, by crossing these mice to mice with targeted disruption of *Pten*, it was shown that the PIN lesions progressed much more rapidly into more advanced PIN and early invasive adenocarcinoma lesions, indicating cooperation between MYC and *Pten* [83]. While we cannot yet determine why there is a discrepancy between these findings by Kim et al. and our findings using Lo-MYC, Hi-MYC, and Super-Lo-MYC, in terms of the issue of whether all cells with MYC overexpression appear morphologically transformed into PIN cells, it is possible that there are mouse strain differences in the ability of MYC to directly transform prostate luminal cells. The study by Kim et al. was carried out on a mixed C57/B6 and 129 genetic background, whereas Lo-MYC, Hi-MYC, Super-Lo-MYC, are all on the FVB genetic background. Further studies that show acute overexpression of MYC in an inducible fashion in different mouse strains may help resolve the differences seen between these results.

### Are Mouse Models of Prostate Cancer That Are Driven by Transgenes under the Control of Androgen Receptor Valid?

One potential criticism of AR-driven transgenic models of prostate cancer is that the system does not reflect the human disease. However, the discovery that a known oncogenic transcription factor (e.g. ERG or ETV1-4) can be driven by AR, as a result of the generation of fusion genes such as *TMPRSS2-ERG* [84], should serve to alleviate such concerns. In fact, Sun et al. have shown that ERG can drive expression of MYC [85]. While this is not proven in humans, it is quite intriguing and further supports the relevance of MYC-based prostate cancer models driven by AR as highly relevant to the human disease. Certainly, additional studies to determine why MYC is highly overexpressed in human PIN and adenocarcinoma lesions are warranted. Given the fact that human high grade PIN and adenocarcinoma lesions typically show a range of proliferative fraction (as indicated for example by Ki67 staining) of between approximately 3–40%, MYC levels in these lesions cannot be elevated simply as a reflection of increased proliferation since in most cases where MYC is overexpressed in human PIN and/or adenocarcinoma, many more cells stain positive for MYC (often 60%–80% of cells stain positive) than could be accounted for by simply increased proliferative fraction [31]. In addition, double-label immunofluorescence in human prostatic adenocarcinoma for MYC and Ki67 reveal a discordance in many of the cells (AM De Marzo, Uzoma Anele, MengMeng Xu, unpublished observations). A recent result may shed light on why MYC is overexpressed in at least some prostate cancers. Wang et al. showed that targeted disruption of *FoxP3* (a gene that is critical to the development of regulatory T cells) in the mouse prostate results in the overexpression of Myc, prostatic hyperplasia, and PIN[86]. Further, they also showed that inactivating deletions or point mutations are fairly common in the *FOXP3* gene in human prostate cancers and PIN lesions [86], and that FOXP3 can directly repress MYC mRNA levels.

In summary, our new data shows that MYC appears to be sufficient to morphologically transform prostate luminal epithelial cells into PIN. Further, we verify that the histopathological features of MYC driven mouse PIN and early adenocarcinoma are highly similar to their human counterparts, and have shown that Nkx3.1 mRNA and protein are negatively regulated in PIN lesions by MYC. Additionally, we identified a time-dependent intermediate step occurring prior to the development of invasive adenocarcinoma in Lo-MYC and Hi-MYC mice. These findings should facilitate studies into molecular alterations, including profound biochemical changes, changes in global chromatin architecture, and changes in the expression of multiple specific genes that are induced by MYC that result in PIN and subsequent invasive adenocarcinoma. Further, these studies support the concept that MYC-based mouse models of prostate cancer are useful “reagent mice” for studying the genetic, epigenetic, and microenvironmental changes that cooperate with MYC and lead to advanced and metastatic disease.

## Supporting Information

Figure S1 Box plot showing increased proliferative fraction in all neoplastic lesions in Lo-MYC mice. Standard slides were immunostained against Ki67 as a marker of cell proliferation. Slides were scanned at 200× magnification using the Aperio Scanscope CS and random snapshot fields were taken. Then, the extent of brown staining (area fraction of Ki67) and blue staining (area fraction of nuclei in question) were identified using FriDA and a ratio of brown/(brown + blue) areas were determined. Each dot in the box plots represents the average ratio from a number of random images (minimum of 3 images per case). p values above box plots indicate comparisons to normal mouse epithelium from age matched FVB mice (Normal).
(0.69 MB TIF)

## References

[1] Ellwood-Yen K, Graeber TG, Wongvipat J, Iruela-Arispe ML, Zhang J (2003). Myc-driven murine prostate cancer shares molecular features with human prostate tumors.. Cancer Cell.

[2] Abate-Shen C, Shen MM (2000). Molecular genetics of prostate cancer.. Genes Dev.

[3] Elo JP, Visakorpi T (2001). Molecular genetics of prostate cancer.. Ann Med.

[4] Nelson WG, De Marzo AM, Isaacs WB (2003). Prostate cancer.. N Engl J Med.

[5] Gonzalgo ML, Isaacs WB (2003). Molecular pathways to prostate cancer.. J Urol.

[6] Allam CK, Bostwick DG, Hayes JA, Upton MP, Wade GG (1996). Interobserver variability in the diagnosis of high-grade prostatic intraepithelial neoplasia and adenocarcinoma.. Mod Pathol.

[7] Bostwick DG, Pacelli A, Lopez-Beltran A (1996). Molecular biology of prostatic intraepithelial neoplasia.. Prostate.

[8] Egevad L, Allsbrook WC, Epstein JI (2006). Current practice of diagnosis and reporting of prostatic intraepithelial neoplasia and glandular atypia among genitourinary pathologists.. Mod Pathol.

[9] Epstein JI (2009). Precursor lesions to prostatic adenocarcinoma.. Virchows Arch.

[10] Leong KG, Wang BE, Johnson L, Gao WQ (2008). Generation of a prostate from a single adult stem cell.. Nature.

[11] Lawson DA, Witte ON (2007). Stem cells in prostate cancer initiation and progression.. J Clin Invest.

[12] Goldstein AS, Lawson DA, Cheng D, Sun W, Garraway IP (2008). Trop2 identifies a subpopulation of murine and human prostate basal cells with stem cell characteristics.. Proc Natl Acad Sci U S A.

[13] Signoretti S, Pires MM, Lindauer M, Horner JW, Grisanzio C (2005). p63 regulates commitment to the prostate cell lineage.. Proc Natl Acad Sci U S A.

[14] Bonkhoff H, Stein U, Remberger K (1994). Multidirectional differentiation in the normal, hyperplastic, and neoplastic human prostate: simultaneous demonstration of cell-specific epithelial markers.. Hum Pathol.

[15] Barclay WW, Axanova LS, Chen W, Romero L, Maund SL (2008). Characterization of adult prostatic progenitor/stem cells exhibiting self-renewal and multilineage differentiation.. Stem Cells.

[16] Blum R, Gupta R, Burger PE, Ontiveros CS, Salm SN (2009). Molecular signatures of prostate stem cells reveal novel signaling pathways and provide insights into prostate cancer.. PLoS One.

[17] Bonkhoff H, Stein U, Remberger K (1994). The proliferative function of basal cells in the normal and hyperplastic human prostate.. Prostate.

[18] McNeal JE, Haillot O, Yemoto C (1995). Cell proliferation in dysplasia of the prostate: analysis by PCNA immunostaining.. Prostate.

[19] De Marzo AM, Nelson WG, Meeker AK, Coffey DS (1998). Stem cell features of benign and malignant prostate epithelial cells.. J Urol.

[20] Meeker AK, Hicks JL, Platz EA, March GE, Bennett CJ (2002). Telomere shortening is an early somatic DNA alteration in human prostate tumorigenesis.. Cancer Res.

[21] De Marzo AM, Meeker AK, Epstein JI, Coffey DS (1998). Prostate stem cell compartments: expression of the cell cycle inhibitor p27Kip1 in normal, hyperplastic, and neoplastic cells.. Am J Pathol.

[22] Verhagen AP, Ramaekers FC, Aalders TW, Schaafsma HE, Debruyne FM (1992). Colocalization of basal and luminal cell-type cytokeratins in human prostate cancer.. Cancer Research.

[23] Nakayama M, Bennett CJ, Hicks JL, Epstein JI, Platz EA (2003). Hypermethylation of the human GSTP1 CpG island is present in a subset of proliferative inflammatory atrophy lesions but not in normal or hyperplastic epithelium of the prostate: a detailed study using Laser-Capture Microdissection.. Am J Pathol.

[24] De Marzo AM, Smearman E, Meeker AK, Iwata T (2007). Telomere shortening in prostate atrophy lesions.. J Urol.

[25] Wang X, Kruithof-de Julio M, Economides KD, Walker D, Yu H (2009). A luminal epithelial stem cell that is a cell of origin for prostate cancer.. Nature.

[26] Nesbit CE, Tersak JM, Prochownik EV (1999). MYC oncogenes and human neoplastic disease.. Oncogene.

[27] Jenkins RB, Qian J, Lieber MM, Bostwick DG (1997). Detection of c-myc oncogene amplification and chromosomal anomalies in metastatic prostatic carcinoma by fluorescence in situ hybridization.. Cancer Res.

[28] Sato K, Qian J, Slezak JM, Lieber MM, Bostwick DG (1999). Clinical significance of alterations of chromosome 8 in high-grade, advanced, nonmetastatic prostate carcinoma.. J Natl Cancer Inst.

[29] Qian J, Jenkins RB, Bostwick DG (1997). Detection of chromosomal anomalies and c-myc gene amplification in the cribriform pattern of prostatic intraepithelial neoplasia and carcinoma by fluorescence in situ hybridization.. Mod Pathol.

[30] Fleming WH, Hamel A, MacDonald R, Ramsey E, Pettigrew NM (1986). Expression of the c-myc protooncogene in human prostatic carcinoma and benign prostatic hyperplasia.. Cancer Res.

[31] Gurel B, Iwata T, Koh CM, Jenkins RB, Lan F (2008). Nuclear MYC protein overexpression is an early alteration in human prostate carcinogenesis.. Mod Pathol.

[32] Zhang X, Lee C, Ng PY, Rubin M, Shabsigh A (2000). Prostatic neoplasia in transgenic mice with prostate-directed overexpression of the c-myc oncoprotein.. Prostate.

[33] Williams K, Fernandez S, Stien X, Ishii K, Love HD (2005). Unopposed c-MYC expression in benign prostatic epithelium causes a cancer phenotype.. Prostate.

[34] Dang CV, O'Donnell KA, Zeller KI, Nguyen T, Osthus RC (2006). The c-Myc target gene network.. Semin Cancer Biol.

[35] Knoepfler PS (2008). Why Myc? An unexpected ingredient in the stem cell cocktail.. Cell Stem Cell.

[36] Meyer N, Penn LZ (2008). Reflecting on 25 years with MYC.. Nat Rev Cancer.

[37] Cotterman R, Jin VX, Krig SR, Lemen JM, Wey A (2008). N-Myc regulates a widespread euchromatic program in the human genome partially independent of its role as a classical transcription factor.. Cancer Res.

[38] Knoepfler PS (2007). Myc goes global: new tricks for an old oncogene.. Cancer Res.

[39] Knoepfler PS, Zhang XY, Cheng PF, Gafken PR, McMahon SB (2006). Myc influences global chromatin structure.. Embo J.

[40] Frese KK, Tuveson DA (2007). Maximizing mouse cancer models.. Nat Rev Cancer.

[41] Gingrich JR, Barrios RJ, Morton RA, Boyce BF, DeMayo FJ (1996). Metastatic prostate cancer in a transgenic mouse.. Cancer Res.

[42] Masumori N, Thomas TZ, Chaurand P, Case T, Paul M (2001). A probasin-large T antigen transgenic mouse line develops prostate adenocarcinoma and neuroendocrine carcinoma with metastatic potential.. Cancer Res.

[43] Huss WJ, Gray DR, Tavakoli K, Marmillion ME, Durham LE (2007). Origin of androgen-insensitive poorly differentiated tumors in the transgenic adenocarcinoma of mouse prostate model.. Neoplasia.

[44] Chiaverotti T, Couto SS, Donjacour A, Mao JH, Nagase H (2008). Dissociation of epithelial and neuroendocrine carcinoma lineages in the transgenic adenocarcinoma of mouse prostate model of prostate cancer.. Am J Pathol.

[45] Pritchard C, Mecham B, Dumpit R, Coleman I, Bhattacharjee M (2009). Conserved gene expression programs integrate mammalian prostate development and tumorigenesis.. Cancer Res.

[46] Hoffman B, Liebermann DA (2008). Apoptotic signaling by c-MYC.. Oncogene.

[47] Freie BW, Eisenman RN (2008). Ratcheting Myc.. Cancer Cell.

[48] Murphy DJ, Junttila MR, Pouyet L, Karnezis A, Shchors K (2008). Distinct thresholds govern Myc's biological output in vivo.. Cancer Cell.

[49] Drayton S, Rowe J, Jones R, Vatcheva R, Cuthbert-Heavens D (2003). Tumor suppressor p16INK4a determines sensitivity of human cells to transformation by cooperating cellular oncogenes.. Cancer Cell.

[50] Gandarillas A, Watt FM (1997). c-Myc promotes differentiation of human epidermal stem cells.. Genes Dev.

[51] Gil J, Kerai P, Lleonart M, Bernard D, Cigudosa JC (2005). Immortalization of primary human prostate epithelial cells by c-Myc.. Cancer Res.

[52] Bhatia-Gaur R, Donjacour AA, Sciavolino PJ, Kim M, Desai N (1999). Roles for Nkx3.1 in prostate development and cancer.. Genes Dev.

[53] Tanaka M, Komuro I, Inagaki H, Jenkins NA, Copeland NG (2000). Nkx3.1, a murine homolog of Ddrosophila bagpipe, regulates epithelial ductal branching and proliferation of the prostate and palatine glands.. Dev Dyn.

[54] Schneider A, Brand T, Zweigerdt R, Arnold H (2000). Targeted disruption of the Nkx3.1 gene in mice results in morphogenetic defects of minor salivary glands: parallels to glandular duct morphogenesis in prostate.. Mech Dev.

[55] Bieberich CJ, Fujita K, He WW, Jay G (1996). Prostate-specific and androgen-dependent expression of a novel homeobox gene.. J Biol Chem.

[56] Bowen C, Bubendorf L, Voeller HJ, Slack R, Willi N (2000). Loss of NKX3.1 expression in human prostate cancers correlates with tumor progression.. Cancer Res.

[57] Bethel CR, Faith D, Li X, Guan B, Hicks JL (2006). Decreased NKX3.1 protein expression in focal prostatic atrophy, prostatic intraepithelial neoplasia and adenocarcinoma: association with Gleason score and chromosome 8p deletion.. Cancer Res.

[58] Asatiani E, Huang WX, Wang A, Rodriguez Ortner E, Cavalli LR (2005). Deletion, methylation, and expression of the NKX3.1 suppressor gene in primary human prostate cancer.. Cancer Res.

[59] Abate-Shen C, Shen MM, Gelmann E (2008). Integrating differentiation and cancer: the Nkx3.1 homeobox gene in prostate organogenesis and carcinogenesis.. Differentiation.

[60] Gordon JW, Scangos GA, Plotkin DJ, Barbosa JA, Ruddle FH (1980). Genetic transformation of mouse embryos by microinjection of purified DNA.. Proc Natl Acad Sci U S A.

[61] Chen H, Mutton LN, Prins GS, Bieberich CJ (2005). Distinct regulatory elements mediate the dynamic expression pattern of Nkx3.1.. Dev Dyn.

[62] Copeland NG, Jenkins NA, Court DL (2001). Recombineering: a powerful new tool for mouse functional genomics.. Nat Rev Genet.

[63] Bradshaw MS, Bollekens JA, Ruddle FH (1995). A new vector for recombination-based cloning of large DNA fragments from yeast artificial chromosomes.. Nucleic Acids Res.

[64] Wang S, Garcia AJ, Wu M, Lawson DA, Witte ON (2006). Pten deletion leads to the expansion of a prostatic stem/progenitor cell subpopulation and tumor initiation.. Proc Natl Acad Sci U S A.

[65] Song H, Zhang B, Watson MA, Humphrey PA, Lim H (2009). Loss of Nkx3.1 leads to the activation of discrete downstream target genes during prostate tumorigenesis.. Oncogene.

[66] Abdulkadir SA, Magee JA, Peters TJ, Kaleem Z, Naughton CK (2002). Conditional loss of nkx3.1 in adult mice induces prostatic intraepithelial neoplasia.. Mol Cell Biol.

[67] Park JH, Walls JE, Galvez JJ, Kim M, Abate-Shen C (2002). Prostatic intraepithelial neoplasia in genetically engineered mice.. Am J Pathol.

[68] Shappell SB, Thomas GV, Roberts RL, Herbert R, Ittmann MM (2004). Prostate pathology of genetically engineered mice: definitions and classification. The consensus report from the Bar Harbor meeting of the Mouse Models of Human Cancer Consortium Prostate Pathology Committee.. Cancer Res.

[69] van Leenders GJ, Gage WR, Hicks JL, van Balken B, Aalders TW (2003). Intermediate cells in human prostate epithelium are enriched in proliferative inflammatory atrophy.. Am J Pathol.

[70] De Marzo AM, Platz EA, Sutcliffe S, Xu J, Gronberg H (2007). Inflammation in prostate carcinogenesis.. Nat Rev Cancer.

[71] Zhang H, Gao P, Fukuda R, Kumar G, Krishnamachary B (2007). HIF-1 inhibits mitochondrial biogenesis and cellular respiration in VHL-deficient renal cell carcinoma by repression of C-MYC activity.. Cancer Cell.

[72] Epstein JI, Amin MB, Reuter VE (2004). Bladder biopsy interpretation..

[73] McNeal JE, Alroy J, Leav I, Redwine EA, Freiha FS (1988). Immunohistochemical evidence for impaired cell differentiation in the premalignant phase of prostate carcinogenesis.. American Journal of Clinical Pathology.

[74] Shen MM, Abate-Shen C (2003). Roles of the Nkx3.1 homeobox gene in prostate organogenesis and carcinogenesis.. Dev Dyn.

[75] Korkmaz CG, Korkmaz KS, Manola J, Xi Z, Risberg B (2004). Analysis of androgen regulated homeobox gene NKX3.1 during prostate carcinogenesis.. J Urol.

[76] Chuang AY, DeMarzo AM, Veltri RW, Sharma RB, Bieberich CJ (2007). Immunohistochemical differentiation of high-grade prostate carcinoma from urothelial carcinoma.. Am J Surg Pathol.

[77] Lei Q, Jiao J, Xin L, Chang CJ, Wang S (2006). NKX3.1 stabilizes p53, inhibits AKT activation, and blocks prostate cancer initiation caused by PTEN loss.. Cancer Cell.

[78] Xu LL, Srikantan V, Sesterhenn IA, Augustus M, Dean R (2000). Expression profile of an androgen regulated prostate specific homeobox gene NKX3.1 in primary prostate cancer.. J Urol.

[79] Markowski MC, Bowen C, Gelmann EP (2008). Inflammatory cytokines induce phosphorylation and ubiquitination of prostate suppressor protein NKX3.1.. Cancer Res.

[80] Li X, Guan B, Maghami S, Bieberich CJ (2006). NKX3.1 is regulated by protein kinase CK2 in prostate tumor cells.. Mol Cell Biol.

[81] West AG, Fraser P (2005). Remote control of gene transcription.. Hum Mol Genet.

[82] Yan Y, Sheppard PC, Kasper S, Lin L, Hoare S (1997). Large fragment of the probasin promoter targets high levels of transgene expression to the prostate of transgenic mice.. Prostate.

[83] Kim J, Eltoum IE, Roh M, Wang J, Abdulkadir SA (2009). Interactions between cells with distinct mutations in c-MYC and Pten in prostate cancer.. PLoS Genet.

[84] Tomlins SA, Rubin MA, Chinnaiyan AM (2006). Integrative biology of prostate cancer progression.. Annu Rev Pathol.

[85] Sun C, Dobi A, Mohamed A, Li H, Thangapazham RL (2008). TMPRSS2-ERG fusion, a common genomic alteration in prostate cancer activates C-MYC and abrogates prostate epithelial differentiation.. Oncogene.

[86] Wang L, Liu R, Li W, Chen C, Katoh H (2009). Somatic single hits inactivate the X-linked tumor suppressor FOXP3 in the prostate.. Cancer Cell.

