# Supplementary figures and images for: MYC Overexpression Induces Prostatic Intraepithelial Neoplasia and Loss of Nkx3.1 in Mouse Luminal Epithelial Cells
Source: PLoS One. 2010 Feb 25;5(2):e9427. doi: 10.1371/journal.pone.0009427 (PMC2828486; doi:10.1371/journal.pone.0009427)

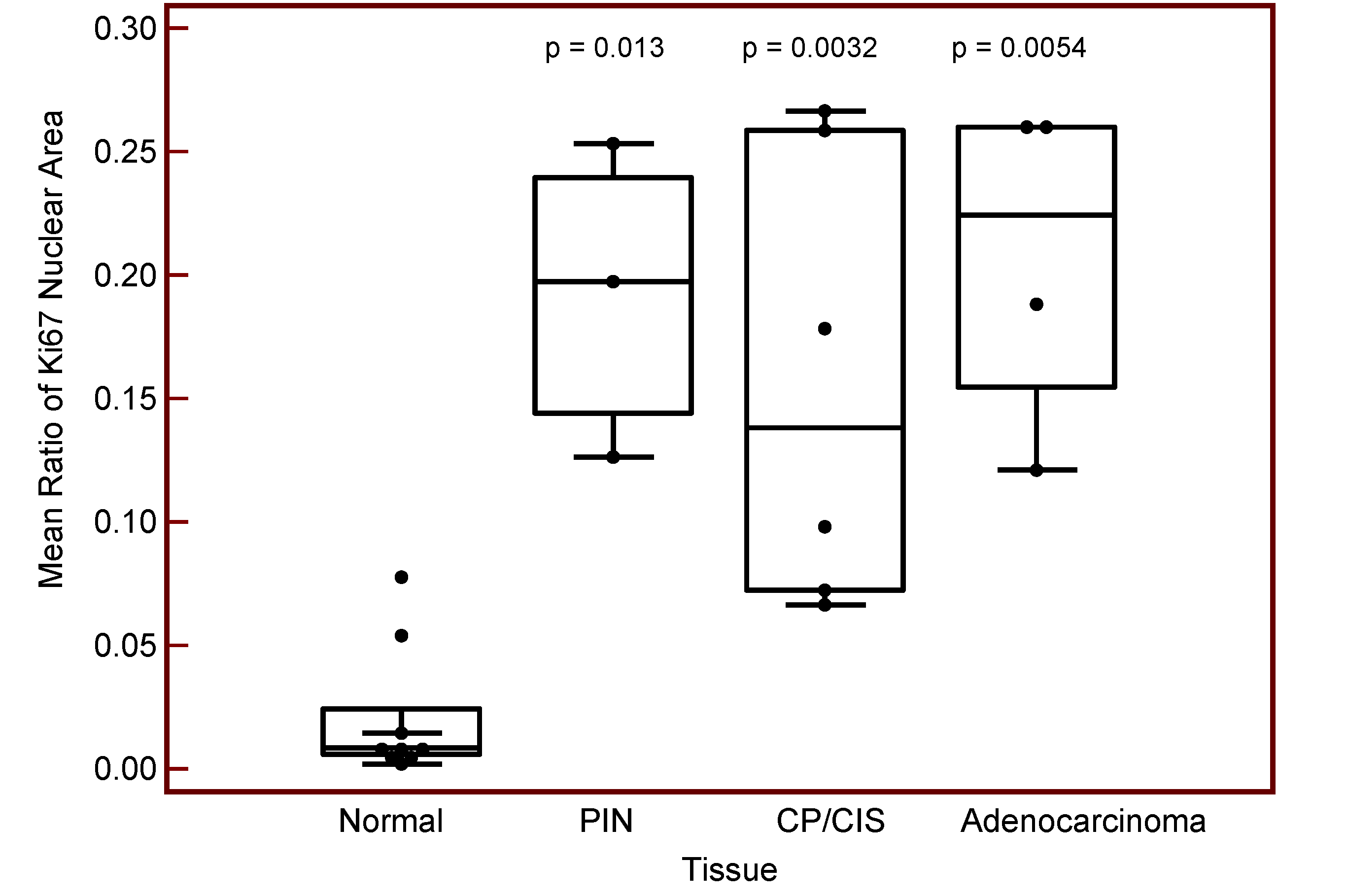

Supplement: Figure S1 — Box plot showing increased proliferative fraction in all neoplastic lesions in Lo-MYC mice. Standard slides were immunostained against Ki67 as a marker of cell proliferation. Slides were scanned at 200× magnification using the Aperio Scanscope CS and random snapshot fields were taken. Then, the extent of brown staining (area fraction of Ki67) and blue staining (area fraction of nuclei in question) were identified using FriDA and a ratio of brown/(brown + blue) areas were determined. Each dot in the box plots represents the average ratio from a number of random images (minimum of 3 images per case). p values above box plots indicate comparisons to normal mouse epithelium from age matched FVB mice (Normal). (0.69 MB TIF) [file pone.0009427.s001.tif]
